# From Contact to Enact: Reducing Prejudice Toward Physical Disability Using Engagement Strategies

**DOI:** 10.3389/fpsyg.2021.602779

**Published:** 2022-01-12

**Authors:** Kristian Moltke Martiny, Helene Scott-Fordsmand, Andreas Rathmann Jensen, Asger Juhl, David Eskelund Nielsen, Thomas Corneliussen

**Affiliations:** ^1^Center for Subjectivity Research, Faculty of Humanities, University of Copenhagen, Copenhagen, Denmark; ^2^Stages of Science, Copenhagen, Denmark; ^3^Enactlab, Copenhagen, Denmark; ^4^Medical Museion, Department of Public Health, University of Copenhagen, Copenhagen, Denmark

**Keywords:** contact hypothesis, second-person cognitive science, prejudice reduction, attitude change, physical disability

## Abstract

The contact hypothesis has dominated work on prejudice reduction and is often described as one of the most successful theories within social psychology. The hypothesis has nevertheless been criticized for not being applicable in real life situations due to unobtainable conditions for direct contact. Several indirect contact suggestions have been developed to solve this “application challenge.” Here, we suggest a hybrid strategy of both direct and indirect contact. Based on the second-person method developed in social psychology and cognition, we suggest working with an engagement strategy as a hybrid hypothesis. We expand on this suggestion through an engagement-based intervention, where we implement the strategy in a theater performance and investigate the effects on prejudicial attitudes toward people with physical disabilities. Based on the results we reformulate our initial engagement strategy into the Enact (Engagement, Nuancing, and Attitude formation) hypothesis. To deal with the application challenge, this hybrid hypothesis posits two necessary conditions for prejudice reduction. Interventions should: (1) work with engagement to reduce prejudice, and (2) focus on the second-order level of attitudes formation. Here the aim of the prejudice reduction is not attitude correction, but instead the nuancing of attitudes.

## Introduction

The contact hypothesis, or intergroup contact theory, is described as one of the most tested, and yet one of the most controversial theories within work on prejudice (Brown, 1988). Since it was formalized by Williams (1947) and Allport (1954), it has been one of the dominant frameworks within work on prejudice reduction. Allport originally defined prejudice as a feeling, favorable or unfavorable, toward a person or thing, that is not based on actual experience (ibid). In a more recent definition, prejudice is described as the negative evaluation of a group or its members based on group membership (Crandall et al., 2002). The basic assumption in the contact hypothesis is that direct contact – under appropriate conditions – will provide opportunity for actual experience and reduce prejudice and negative evaluations between in- and out-groups and their members.

The contact hypothesis still enjoys immense research attention, and a variety of studies provide solid empirical confirmation that contact does in fact reduce prejudice (Pettigrew and Tropp, 2006; Page-Gould et al., 2008; Binder et al., 2009; Vezzali et al., 2010; Davies et al., 2011; Swart et al., 2011; Mickus and Bowen, 2017; Paluck et al., 2018; De Coninck et al., 2020). In order to control the quality of the contact and avoid the risk of counterproductive contact (e.g., strengthening stereotypes), the contact hypothesis operates with a set of structural conditions. Since Allport (1954) original formulation of four basic conditions (equal status, common goals, intergroup cooperation, and support of authorities, law or customs), the list of conditions has expanded throughout the years.

Already by 1997, Wright et al. compared the contact hypothesis to a grocery list solution. Following Pettigrew’s (1998) argument that researchers have overburdened the hypothesis with too many conditions, Dixon et al. (2005) collected a list of 13 conditions based on the available literature and highlighted the narrow academic focus that the theory has attracted. They re-named it the “optimal contact strategy” and pointed out that in aiming to avoid backlash, the contact hypothesis has become an idealist aim of optimizing conditions rather than application. This aim, along with the fact that contact in everyday life is not always possible or may even be resisted because of the prejudice it is aimed at reducing (Vezzali et al., 2014), has put the contact hypothesis in danger of becoming untranslatable to any real-life situations. Further, Paluck et al. (2018) point out that only a minority of studies succeed in ensuring just the four conditions originally given by Allport.

A range of suggestions already exist for modifying and/or extending the contact hypothesis to make it more applicable in real-life situations, addressing what can be called the “application challenge.” Some of the most influential suggestions share a strategy of moving toward forms of *indirect* contact through either acquaintance, media, technology or imagination, as seen in the *Extended* (Wright et al., 1997), the *Vicarious* (Gómez and Huici, 2008) the *Para-social* (Horton and Wohl, 1956; Schiappa et al., 2005), the *Imagined* (Crisp and Turner, 2009) or the *Electronic* or *E-contact* (Amichai-Hamburger and McKenna, 2006; White et al., 2015) contact hypothesis. These indirect approaches have been widely published on, and all have shown evidence of positive effect (Gómez and Huici, 2008; Davies et al., 2011; Lemmer and Wagner, 2015; Zhou et al., 2019).

The main challenge that these proposals try to solve by way of indirectness, is how to negotiate real-world applicability while avoiding the risk of a counterproductive effect of contact. Many of them share the strategy of minimizing intergroup anxiety, i.e., anticipated negative reactions or consequences of an intergroup encounter (Stephan and Stephan, 1985, 2001). A reduction in intergroup anxiety is known to be a significant factor in increasing the effect of contact as means to reduce prejudice (Stephan and Stephan, 1985; Dijker, 1987; Islam and Hewstone, 1993; Stephan et al., 2002; Voci and Hewstone, 2003; Paolini et al., 2004; Brown and Hewstone, 2005). It is suggested that the mechanism behind such effect is that intergroup anxiety creates a disengagement with the other person. As a consequence, this makes individuals rely more heavily on pre-existing stereotypes, causing them to jump to stereotypical conclusions, and making them pay less attention to counter-stereotypical behavior (Wilder and Shapiro, 1989; Wilder, 1993).

In the following, we propose to deal with the “application challenge” in a hybrid way that includes and combines aspects of both direct and indirect contact strategies. We aim to see if it is possible to maintain aspects of the embodied contact from the direct contact hypothesis, while at the same time use the indirect strategy to minimize intergroup anxiety and counteract the development of disengagement. We develop this hybrid strategy by drawing on the second-person method in cognitive science and its focus on engagement.

### A Second-Person Method: Engagement Strategy

Within the past 20 years currents in social psychology and cognitive science have turned their attention toward an understanding of cognition as embodied, enacted, emotive, extended and embedded (Varela et al., 2000; Gallagher, 2005; Chemero, 2009; Rowlands, 2010; Stewart et al., 2010; Ward and Stapleton, 2012; Colombetti, 2014). This trend is especially prominent in the Interactive Turn (de Jaegher et al., 2010) in social cognition, which suggests using a second-person methodology when exploring social cognitive phenomena (Thompson, 2001). This turn emphasizes the importance of both engagement and reciprocal embodied interaction (e.g., de Bruin et al., 2012; Schilbach et al., 2013; Satne and Roepstorff, 2015), the overall idea being that these features of social psychology and cognition are necessary to consider when researching social phenomena such as prejudice reduction or attitude change.

Satne and Roepstorff (2015) define engagement as involving two aspects: (1) an experiential aspect, and (2) a normative aspect. The first aspect illustrates that there is an inherent experiential trait in entering into relations of engagement with others, which can occur in many different relations such as: bodily interactions (e.g., dancing), collaborations (e.g., moving a couch together) or a shared experience (e.g., watching a movie together). The experiential trait is an affective, emotional, and reciprocal we-experience. However, as the second aspect emphasizes, such experience and feeling of the other has a normative trait. The experience is one in which I commit to the other as a person, and vice versa. The relation of engagement is thus structured through a mutual and personal commitment. That is: bodily interaction, collaboration and shared experience can drive mutual commitment and engagement.

Within the contact hypothesis literature, we find a similar notion in Tropp and Barlow (2018). They argue that contact in the contact hypothesis is effective if and when it encourages what they call “psychological investment.” Such investment is connected to notions of empathy, personal relevance, and humanization. Similar trends can be found in Vezzali et al. (2010) regarding the role of contact in increased empathy and reduced anxiety, and in Capozza et al. (2014) regarding contact and humanness attribution.

Focusing on the positive trait of creating engagement rather than on the negative trait of reducing intergroup-anxiety, we integrate lessons from the second-person methodology, in order to design an intervention which allows us to examine the effect of such engagement strategy on prejudice reduction.

### Engagement Through Theater Performance

In line with other earlier sociocultural psychologists and sociologists the work of Ulric Neisser criticizes research in social psychology for being over-academic and inapplicable in real-life. Neisser suggests that social scientists could benefit from collaborating with professionals from the world of theater, since the expertise of such professions consists of manipulating social impressions (Neisser, 1980). We follow this suggestion and turn to theater in order to design an engagement-based intervention.

Research in theater and performance studies suggests that we can understand theater performance in a way that is very much in line with a second-person methodology. Specifically, it focuses on three notions to understand theater performance: (1) embodiment, (2) engagement, and (3) transformation (e.g., Zarrilli, 2004; McConachie and Hart, 2006; Cook, 2007; McConachie, 2007, 2008; Blair, 2008; Di Benedetto, 2010; Nicholson, 2011; Shaughnessy, 2012).

The notion of “embodiment” in theater and performance studies is used as a way of understanding the “affective” impacts that the performance has on the audience (Thompson, 2009; Nicholson, 2011), where the audience experiences “being kinesthetically moved” (Fenemore, 2003). This “moving” of the audience is due to the felt, embodied experience of the live performance, of being in a shared space and of being haptically affected. Such embodiment leads to an empathic engagement (Shaughnessy, 2012). The notion of “engagement” is therefore already implied in the notion of embodiment, and is used to emphasize that, in theater performance, the distinctions between creator/performer/perceiver are blurred, as performers and audience are in a shared, participatory, and immersed dialogue (Shepherd, 2006; Shaughnessy, 2012). These elements strongly suggest that theater can provide the engagement we aim at in our intervention. Depending on the type of theater performance one can operate with different degrees of embodied engagement, ranging from direct bodily interactions and collaboration between performers and audience to more indirect, sedentary behavior of the audience, as they watch the performers. In the latter case, the theater performance, should, however, still be seen as an embodied, shared and participatory dialogue between performer and audience, as audience and performers still share the same space and are haptically moved and affected by each-other.

The idea and notion of “transformation” is understood by perceiving theater as an encounter with the social community (Taylor, 2003) or as a public and social event (Thompson and Schechner, 2004). The participatory, engaging performance is then used to “effect” social change through its embodied “affect” (Nicholson, 2005; Thompson, 2009), whereby the audience members become “active producers,” rather than consumers, and are transformed with the production of the performance.

Thus, theater affords embodied engagement, both experientially and normatively, and it has the potential to transform social notions, and hence, be the foundation for possible prejudice reduction through attitude change. That is, the format of a performance as well as the engagement “on stage” (i.e., between performers) may act as mediators, much like the indirect strategies, without losing embodiment. Additionally, theater, like many of the indirect strategies, also has the advantage that large numbers of audience can be involved. Theater therefore seems to be a good fit for developing an engagement-based intervention of hybrid contact.

### Changing Attitudes Toward People With Physical Disability

To develop and investigate the possible effects of an engagement-based intervention we focus on the reduction of prejudice toward people with physical disability. Physical disability is a constructive attitude object to work with in an intervention study, since group salience is often immediately present in the encounter, particularly when the disability is visible.

The feelings that people without disability immediately experience toward people with physical disability are typically that of discomfort and fear (Krahé and Altwasser, 2006; Coleman et al., 2015). These feelings arise because people without physical disability are irrationally concerned with “catching” the disability (Park et al., 2003) and they perceive people with disability as being unpredictable, incompetent, weak, dependent on others and lacking strength and endurance (Louvet, 2007; Rohmer and Louvet, 2009, 2012; Novak et al., 2011; Martiny, 2015).

These stereotypical attitudes toward people with physical disability lead to a range of reactions including: bodily objectification and “intersubjective oppression” in everyday face-to-face interactions (Toro, 2020), discrimination and marginalization in the labor marked (Louvet, 2007; Grue, 2016) and in healthcare settings (Toriello et al., 2007; Sterkenburg and Vacaru, 2018) and challenges in forming social relations such as friendships, which has been related to the fact that people without disability also express sadness and pity toward people with disability (Harper, 1999; Lightfoot et al., 1999; Weiserbs and Gottlieb, 2000; Green, 2003, 2007).

The overall aim of the study is to see if an engagement-based intervention can change such prejudicial attitudes toward people with physical disability.

## Method: Changing Attitudes With an Engagement-Based Theater Intervention

**FIGURE 1:**
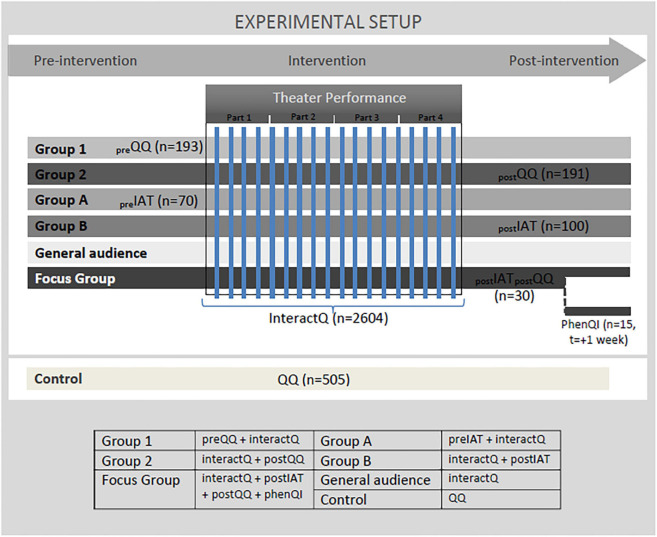
The figure shows the study design with the different test-methods used before (QQ, IAT), during (interactQ) and after (QQ, IAT, phenQI) the performance and the different test-groups that was involved.

The experimental setup was developed around a theater intervention. The study design consisted of tests pre- and post-intervention (quantitative questionnaire, IAT test and qualitative interview) monitoring during intervention (interactive questionnaire), as well as tests of a population control-group (quantitative questionnaire). We outline the experimental setup in more detail in the section “Experimental Instruments: Mixing Methods” (see also Figure 1).

We aimed to create embodied, engaged contact between in-group (i.e., audience) and out-group (i.e., people with physical disability, present on stage), and to test the effect of such contact on attitudes toward people with disability. Our hypothesis was that engagement during the theater performance would reduce prejudice by changing the negative attitudes of participants to more positive attitudes toward people with physical disabilities. The intervention therefore operates with an engagement condition, and the experimental variables measured were the level of engagement during the performance and the change in attitudes toward physical disability before and after the performance.

### The Theater Intervention: Direct and Indirect Contact

The theater performance was set up as a 1 h and 45 min autobiographical stage performance about a 28-year-old man, JN, who lives with quadriplegic cerebral palsy (CP) and has a speech impediment, thus displaying group salience both visibly and audibly at first encounter. The performance would provide embodied engagement with JN (an out-group member) on stage, but was designed with a seated audience, that is, there was no bodily interaction between JN and audience. Instead, and in-group member (i.e., a person without physical disability) interacted with JN on stage, adding a mediated indirect contact to the embodied engagement already present in theater performance.

The performance was developed in close collaboration and conversation between the research group, the theater group, and JN, in order to obtain an accurate image of life with CP. To present a complex life with CP, the performance was divided into four parts. The first, serving as a general introduction to “the person JN,” and the following three dealing with sensitive issues related to life with physical disabilities, i.e., (1) introduction, (2) employment, (3) finding a partner, and (4) parenthood. The autobiographical format was chosen as a way of articulating, exploring, and interrogating a person, identity, and subject through embodied and engaged theatrical strategies. To emphasize both group salience and personalization (see Miller, 2002) documentation material from JN’s “real life” was used in the performance to illustrate the difference between JN’s own perspective and that of science and society. For example, in part 1, JN described the experience of living with congenital brain damage while, in the same part, audio/visual documentation presented a neurologist describing JN’s brain damage based on an MRI scan of JN’s brain. As another example, in part 2, JN’s personal medical journal and his diagnosis were presented and compared to medical and diagnostic demographic statistics of CP in the Danish society (taken from Michelsen, 2006). In the same part, the audience was presented with recordings of JN’s real-life phone conversations for job-interviews, while being informed about statistics of employment, also of people with CP in the Danish society.

JN was the main performer, but he was joined on stage by a non-CP actor (KF), who guided the audience through JN’s life. This was done for practical reasons: to support JN in performing the theater performance, and to help the audience understand JN. The relation between JN and KF was used explicitly to stress their difference (in- and out-group salience), the complexities of this difference (personalization), and as an indirect contact strategy to provide a way for the audience to interact vicariously with JN.

The scenography was minimally designed (see Picture 1) and the lightning, sound, and music were designed to support and enhance the connection between JN and the audience – forming specific moods and helping the varying “real-life” locations appear present during the performance.

The intervention was carried out in two versions, a pilot followed by a larger experiment-set. We conducted the pilot-experiment with a single theater performance and approximately 1,100 people in the audience^1^. The actual experiment was conducted during 17 theater performances, held over the course of a month, with approximately 2,600 people in the audience in total.

**PICTURE 1:**
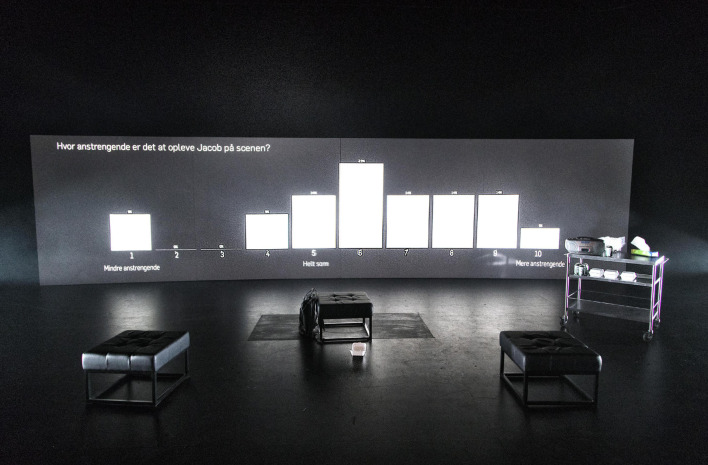
Shows the stage and scenography, where the main prop is an 11 m × 2.5 m projection wall used for an interactive questionnaire (described in section “Experimental Instruments: Mixing Methods”). Other props included three small stools, one leather stool with a connected small table, and two bar stools.

The intervention was carried out in two versions, a pilot followed by a larger experiment-set. We conducted the pilot-experiment with a single theater performance and approximately 1,100 people in the audience^1^. The actual experiment was conducted during 17 theater performances, held over the course of a month, with approximately 2,600 people in the audience in total.

### Participants

The intervention and experimental design were tested and adjusted during the pilot-experiment and was then used in the experiment to test different groups of the total audience sample (*n* = 2,604). Everyone in the audience was given the interactive questionnaire during the performance, but out of the total audience sample, a pre-group (group 1, *n* = 193) that was about to see the performance, and a post-group (group 2, *n* = 191) that had just seen the performance, was given the quantitative questionnaire. A control-group (control, *n* = 505) of Danish citizens (age 18–60) that did not see the performance, where also given the quantitative questionnaire. A pre-group (group A, *n* = 70) and post-group (group B, *n* = 100) were given the IAT tests before and after the performance, respectively. A focus-group of Danish citizens (focus group, *n* = 30; 15 women, 15 men, age 22–59) was given the quantitative questionnaire and the IAT test after the performance, and we also conducted qualitative interviews (phenQI) after the performance.

The audience was informed at the beginning of the performance that the performance was part of an experiment and that they would be given an interactive questionnaire during the performance. They were free not to answer, if they did not wish to participate in the study. Written informed consent was obtained from all individual participants that took part in measurements before and after the performance^2^.

The pre-group (group 1 and group A in Figure 1) and post-group (group 2 and group B in Figure 1) were recruited by random selection from the audience in attendance at the theater across the 17 shows, i.e., people who had come to the performance by own initiative. The test-selected groups were recruited by research and theater staff asking audience members in the theater lobby before the performance and coming out after the performance if they wanted to participate in the research. Random selection was approximated by asking whoever passed once a conversation with one audience member ended. To ensure that changes were not due to increased acquaintance with the questionnaire, none of the individuals in the pre-group and post-group were identical. The quantitative questionnaire was filled out for pre- and post-group at a restricted research test-area close to the theater stage, the same was the case for the IAT test.

To be able to balance biased selection in recruiting at the theater, we recruited a focus-group, as well as a control-group, outside the group who came to the theater on own accord. The focus-group was recruited 2 weeks in advance of the specific performance they were going to see, which was either a performance in the beginning (*n* = 10), middle (*n* = 10) or end (*n* = 10) of the playing period. They were recruited randomly by the research group by selecting people between the ages of 18 and 60 who were walking past the Royal Theater (i.e., on a central square in the capital). Participants were recruited through the following process: after being informed that they would be participating in a research project by accepting two free tickets to a theater show, those who still chose to participate were formally invited into the experiment over an e-mail a few days after in-person recruitment, giving them details of time and place. They were not informed of the kind of research project and topic, but only told that they were going to see a theater performance and would have to do tests after. Out of 70 persons recruited (i.e., who agreed and were sent an e-mail), 30 persons came to the performance. The control-group, who did not see the performance, was recruited randomly online by the research assisting organization Enalyzer, selected based on the same criteria as the focus group and aiming for a balanced distribution in age and gender. We will discuss the participant selection further in the strengths and limitations section.

### Experimental Instruments: Mixing Methods

Previous research on attitudes toward physical disability emphasize the methodological challenge of relying on explicit, self-reported attitude measures, such as the challenge of potential bias through social desirability – where participants answer in a way, they believe is the most socially appropriate (Antonak and Livneh, 2000; Wilson and Scior, 2014). For the experiment we therefore used a mixed-method approach consisting of both explicit (self-reporting) quantitative and qualitative methods, as well as implicit (behavioral) measures.

We specifically used a quantitative questionnaire (QQ) pre- and post-intervention; Implicit Association Test (IAT) pre- and post-intervention; an interactive questionnaire (interactQ) during the intervention; and phenomenological qualitative interviews (phenQI) post-intervention. An overview of all of the study design and the following details can be found in Figure 1.

The mixed method used is phenomenologically framed, which means there is certain credence given to the qualitative method of the data generation and analysis. This is not to downplay the quantitative method, but rather to give it a specific purpose; the purpose being to accompany the qualitative method in gaining different (complementary or divergent) perspectives on the same phenomenon of prejudice toward people with disability^3^.

#### Quantitative Questionnaire (QQ)

The first explicit method used is a 7-point Likert Scale quantitative questionnaire (QQ), to acquire self-reports on attitudes toward people with physical disability. This questionnaire included a set of introductory questions to gather biographical information regarding the participants age, gender/sex, employment, education, and whether they themselves live with a physical disability or have a family member, friend or colleague who lives with physical disability.

In addition to the biographical questions, the questionnaire contained 26 items. Quantitative questionnaires on physical disability used in large-scale, national surveys on the British and Danish perception and attitude toward people with physical disability (Olsen, 2000; Staniland, 2011) already exist. So the questionnaire items was developed by selecting the questions from the previous questionnaires that made sense in relation to both the themes of the theater performance (e.g., employment, relationship and parenthood) and two standardized parameters of prejudice toward physical disability (Coleman et al., 2015): (1) incompetence, that is, the perceived level of competence or lack of competence of a person with physical disability, often in specific tasks; and (2) social distance, that is, the level of intimacy a person is willing to have with another person, e.g., recognize, live near and associate with them (Toriello et al., 2007; Ouellette-Kuntz et al., 2010). Measuring the amount of social distance through the degree of (un)comfortable experience in having relations with people with physical disability will signalize prejudice (Abrams et al., 1990). After selection, the questions were related specifically to CP, making them more specific toward a particular disability than the previous quantitative questionnaires.

The 26 items were randomly mixed between items relating to incompetence and social distance regarding people with CP. Questions of incompetence were framed in relation to work, politics, taking care of children and being independent. Examples of questions (translated from Danish) are: “How productive do you consider a person with CP to be in a work context?”, “How would you feel about a person with CP having a child?”, and “How would you feel if a person with CP took up an important political position in your municipality?”. Questions of social distance were framed in relation to being a friend, partner, or colleague to a person with CP, sitting next to them in public transport, and associating with them in different social settings. Examples of these questions are: “How would you feel about being friends with a person with CP?”, “How would you feel about being served in a store by a person with CP?”, “How would you feel about having a person with CP as a colleague?”. Most questions (all questions on social distance and several of the questions on incompetence) were answered on a scale going from 1 (very uncomfortable) to 7 (very comfortable). The remaining questions of incompetence were answered by (dis)agreeing with posed statements, on a scale going from 1 (strongly disagree) to 7 (strongly agree), or on a scale of productivity going from 1 (very unproductive) to 7 (very productive). Questions were posed so that it varied whether agree/disagree would indicate negative attitudes toward people with disability, so that participants would not score positive or negative as an effect of placing themselves on the same point of the scale.

#### Implicit Association Test (IAT)

The first implicit measurement was the standardized Implicit Association Test (IAT) for measuring implicit attitudes toward physical disability (Greenwald and Banaji, 1995; Greenwald et al., 1998, 2003). The test is a latency-based method that indirectly measures strengths of associations between, in our case, people with and without disability and attributes of either pleasant (good) or unpleasant (bad) valence. The IAT test divides results into a (1) slight, (2) moderate or (3) strong automatic preference for *abled people* compared to *disabled people*; (4) a little to no automatic preference between *abled* and *disabled people*; a (5) slight, (6) moderate or (7) strong automatic preference for *disabled people* compared to *abled people*, and (8) too many errors to determine a result.

The IAT test can be taken online on the topic of physical disability, and the test was used to explicate the audience’s implicit attitudes before and after the intervention. For controls, we relied on accumulated statistical material from regular use of the IAT test. The test was taken at a restricted research test-area close to the theater stage.

#### Interactive Questionnaire (interactQ)

The second implicit measurement was the interactive questionnaire (interactQ), which was developed as a proxy-measure of the audiences’ level of engagement throughout the performance and their behavior in forming their attitude toward physical disability in general and JN specifically. This was done by measuring the consistency in time to answer the question, the number of audience-members who answered, and whether they made answer-revisions.

The interactQ was made up of 18 questions structured within four questioning-sections to coordinate with the four parts of the theater performance. The questions were framed in a way to make the audience reflect on the experiences of living with physical disability and particularly on JN’s experiences. The first section of questions related to the audience’s understanding of physical disability in general, such as “To what degree are you able to familiarize yourself with living with a physical disability?”. The questions in section 2–4 were designed based on concrete theatrical scenes and situations that the audience experienced and shared with JN. The aim was for the audience to reflect on the shared experience, by asking them to either: (1) relate themselves to JN, e.g., “Where would you place your quality of life relative to JN?” or (2) try to answer from JN’s perspective, e.g., “Where do you believe that JN would place his quality of life relative to yours?”.

For each question, the theater performance paused, and the question was announced by a speaker and posed for a time-period of 12 s on the projection wall on the stage (see Picture 1). The answers were submitted using a mobile answering-device placed in the participants’ seats. For technical reasons, i.e., the button function of the device, these questions were answered on a 10-point Likert scale. As the aim of this questionnaire was not the given answers, but the measure of time and behavior of audience in answering, we used different scale labels that corresponded with the performance, ranging from 1 (to the lowest degree) to 10 (to the highest degree), 1 (much worse) to 10 (much better), and 1 (much lower) to 10 (much higher). There was no neutral answer (e.g., I don’t know), and no medium point on the scale, making it necessary for the audience to form their attitude in relation to the specific question. Within the 12 s, participants could revise their answer as many times as they wanted.

When the audience answered, their answers were shown anonymously in real-time below the question on the projection wall on stage (see Picture 1). This aspect, as well as the option to change one’s answer as many times as one wanted, was made clear to the audience at the beginning of the performance. It was not announced that we would be measuring the time it took for the audience members to answer and whether they changed their answers during the time-period.

#### Phenomenological Qualitative Interview (phenQI)

The second explicit method used was semi-structured qualitative interviews, conducted phenomenologically (phenQI) with the focus-group. The interviews were used to understand the quality of the contact with JN.

The focus-group filled out the QQ and took an IAT test after the performance. After each performance, an email was sent to the 30 participants, respectively, asking them if they wanted to participate in a follow-up interview. 15 out of the 30 (5 from each sub-group) agreed to participate, and the follow-up phenQI were conducted 1 week after the performance that they watched. The interviews lasted around 1–11/2 h.

The phenQI used a specific type of interview based on philosophical phenomenology with its own specific second-person questioning and analysis techniques (Høffding and Martiny, 2016; Martiny, 2017; Zahavi and Martiny, 2019). The method is developed particularly to gain insight into second-person experiences. The method employs open “how” questions to draw the attention of the interviewees to detailed pre-reflective aspects of their experience. In our case, the focus was on *how* the audience experienced JN on the stage, specifically in relation to their experience of being in direct and indirect contact with him, their perception of him and the potential change in their perception during the performance. We also asked about their experience of the theater performance in general and their thoughts on their individual QQ, IAT and interactQ test results.

### Analysis: Phenomenological Framing and Triangulation

In mixed method research, one can mix the methods at different stages, e.g., in the data generation, analysis or interpretation. In this study the methods are mixed in the interpretation of the data by using the phenomenologically based second-person theory and understanding of engagement (presented in section “A Second-Person Method: Engagement Strategy”) to combine the quantitative and qualitative datasets into one account of prejudice reduction.

This means that the quantitative and qualitative data were generated and analyzed separately. The quantitative analysis concerned statistical comparison of the data before, during, and after the performance. In relation to the QQ the analysis focused on pre/post differences within the individual questions, mapping the concrete aspects and situations within which attitudes on incompetence and social distance changed. A series of Kruskal-Wallis *H* tests were conducted to determine differences in attitude toward people with physical disability based on a ranking of the responses to the individual questions – with a higher rank indicating a more positive attitude. We did not look at single index or attempt to create factors across the 26 items.

For the data of the IAT test an independent-samples Mann-Whitney *U*-test was conducted in SPSS based on ranking of the results to test for pre-IAT to post-IAT differences. The data of the interactQ was analyzed descriptively, as the technical equipment did not allow data gathering that fulfilled statistical assumptions of independence.

The qualitative data of the phenQI, i.e., the recorded interviews, were transcribed ad verbatim and through multiple rounds of labeling, categorization and repeated listening the data was coded and structured in order to identify experiential categories. The analysis was conducted in accordance with phenomenological methods of descriptive analysis (see Gallagher and Zahavi, 2008, chapter 2), and the strategy of “phenomenological consistency” was employed in order to validate the descriptions (Høffding and Martiny, 2016; Martiny et al., 2021).

After the separate qualitative and quantitative analyses were completed, they were triangulated and mixed into one interpretation (see Discussion). This was done by first providing tentative interpretations of the separate analyses regarding the level of engagement, change in attitudes and possible prejudice reduction toward physical disability that occurred because of the intervention. These tentative interpretations and answers to the research questions were then compared and combined with each other and integrated into one meta-interpretation that also explained the contrast and differences between the tentative interpretations and answers^4^.

## Results

Table 1 presents the results of the biographical questions in the QQ for both test selected audience groups (group 1, 2, A, B, and focus-group) and control group, seen in relation to theater audience in Denmark and general population.

**TABLE 1:** Biographical information: The table shows biographical data for test selected audience groups and control group, as well as data on average theater audience in Denmark and general population.

Biographical information	IAT/QQ (*n* = 554)	Focus (*n* = 30)	Control group (*n* = 505)	Theater audience*	National
Male	65.30%	33.30%	56.50%	40.4%^(1)^	49.8%^(2)^
Female	34.70%	66.70%	43.50%	59.5%^(1)^	50.2%^(2)^
Age (average)	35 years	33 years	39 years	Median: 53/41	41.7^(2)^
**Education**					
Elementary/High School/Vocational education	15.20%	20%	N/A	37.7% ^(1)^	65%^(2)^
Short higher education (2 years)	23.60%	40%	N/A	**	5%^(2)^
Medium higher education and bachelor (2–4 years)	25.60%	0%	N/A	41.5%^(1)^	17%^(2)^
Long higher education (more than 5 years)	35.60%	40%	N/A	20.8%^(1)^	9.9%^(2)^
**Disability relations**					
People with disability	14.70%	6.70%	N/A	N/A	10–15%^(3)^
People who knows a person with disability	45.30%	47.10%	N/A	N/A	N/A

*Biographical data are given for gender, age, education level and disability as well as acquaintance with people with disability.*

* The report uses four categories, where two consist of different theater audiences (positive and negative, respectively) and two of people who have never been to the theater (not relevant for this comparison). Data here represent a weighted sum of the two theater audience group giving us the average theater audience. This explains the double median value. ** Short higher education is included in the medium higher education percentage.

*^(1)^ Rambøl and Applaus, 2020.*

*^(2)^ Denmark’s statistics (Mainz Sørensen, 2019; Mackie, 2020).*

*^(3)^ Rode Larsen and Høgelund, 2015.*

**FIGURE 2:**
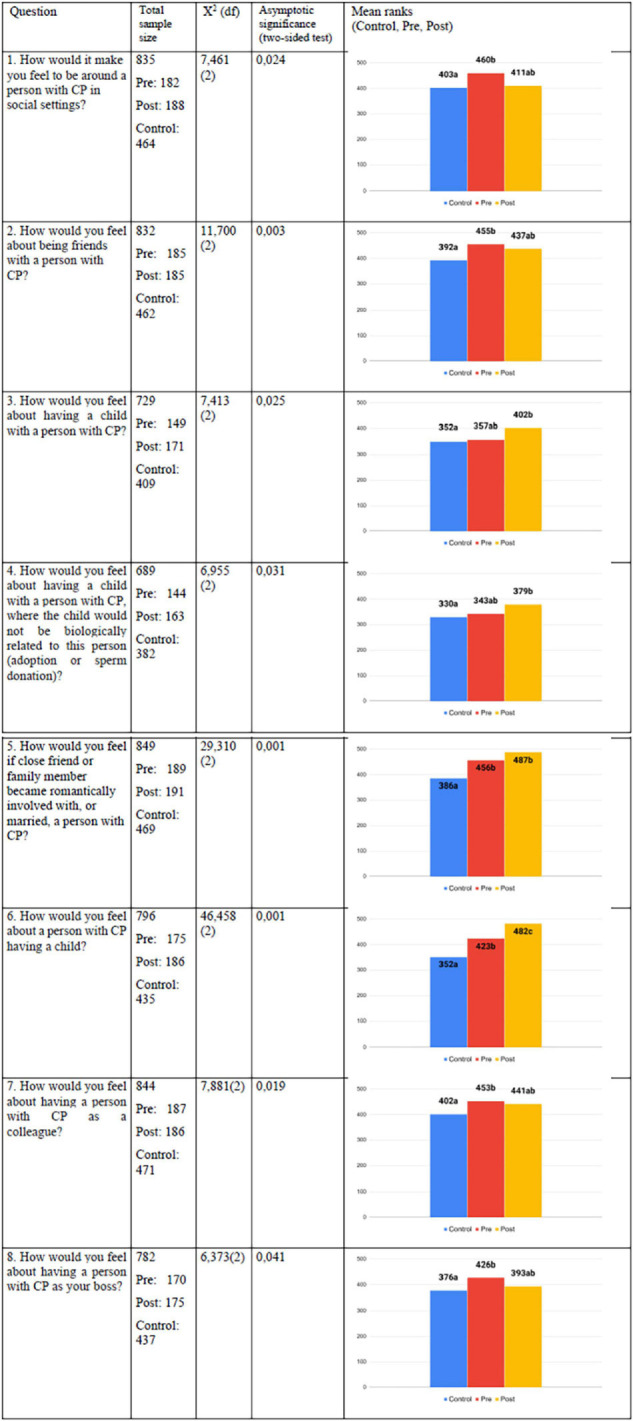
Quantitative questionnaire (QQ). The table shows, from left to right: the question posed, the total sample size, the *X*^2^ values obtained and the degrees of freedom, the asymptotic significance levels based on the *X*^2^ values, a graphing of the mean ranks for the control, pre-QQ and post-QQ groups and identical letters denote no significant difference between mean ranks and non-identical letters denote a significant difference between mean ranks. Total sample sizes differ between 689 and 849 respondents due to both missing answers to the questions and difference in responses of “Do not know,” which were excluded in the statistical analysis.

Figure 2 presents a selected sample of the questions in the QQ that showed significant results^5^. Out of the 26 QQ questions, 8 questions (renamed as questions Q1-Q8) showed significant differences (*P* < 0.05) between various groups regarding concrete attitude changes to physical disability. When compared to the control-group, the post-QQ participants’ attitudes are more positive in their evaluations of questions on social distance (e.g., about having a relationship with people with CP) and on competence (e.g., their ability to become parents and care for a child). For the questions Q3, Q4, Q5, and Q6 the post-intervention group (group 2) differ significantly from the control-group.

For these questions, post-intervention replies had the highest mean rank in terms of positive attitudes toward people with CP, the pre-QQ group (group 1) had the medium mean rank and the control-group had the lowest mean rank. For questions Q3 and Q4 no other differences were significant. For question Q5 the control-group scored significantly lower than both pre- and post-intervention group (group 1 and 2). For question Q6 all three groups scored significantly different in terms of attitudes toward people with CP where control group had the lowest, pre-group medium and post-group the highest mean rank (group 1). For the questions Q1, Q2, Q7 and Q8 the pre-QQ group (group 1) differ significantly from the control-group, but no other differences were significant. This concerns social distance in relation to being friends with people with CP, and competence in regard to employment, i.e., working with people with CP. For these questions, group 1 has the highest mean rank in terms of positive attitudes toward people with CP, the control-group had the lowest mean rank, with post-intervention group (group 2) scoring in-between, not being significantly different from either of the two other groups.

**FIGURE 3:**
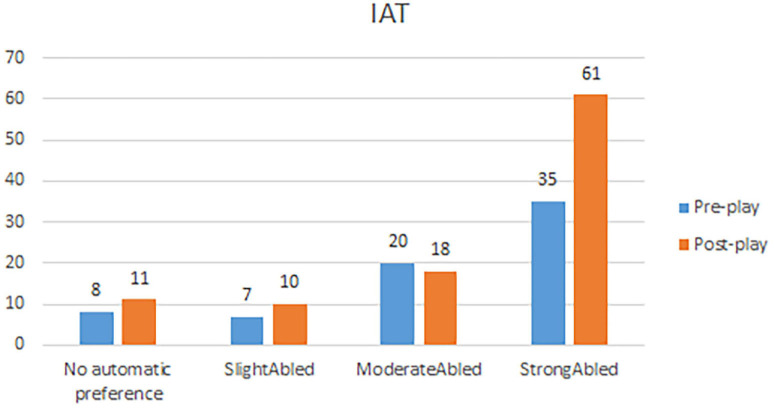
Implicit Association Test (IAT). The figure shows the distribution of results on the IAT for the pre-IAT (*n* = 70) and the post-IAT (*n* = 100) group.

Figure 3 shows the results of the IAT test of the pre-IAT (group A) and post-IAT (group B), ranging from “no automatic preference for abled or disabled people” to a “strong preference for abled people over disabled^6^.” The data was ranked with a higher rank, representing a stronger preference for abled people over disabled people. The test showed no significant changes at the implicit attitude level between pre- and post-group (*P* = 0.273).

**FIGURE 4:**
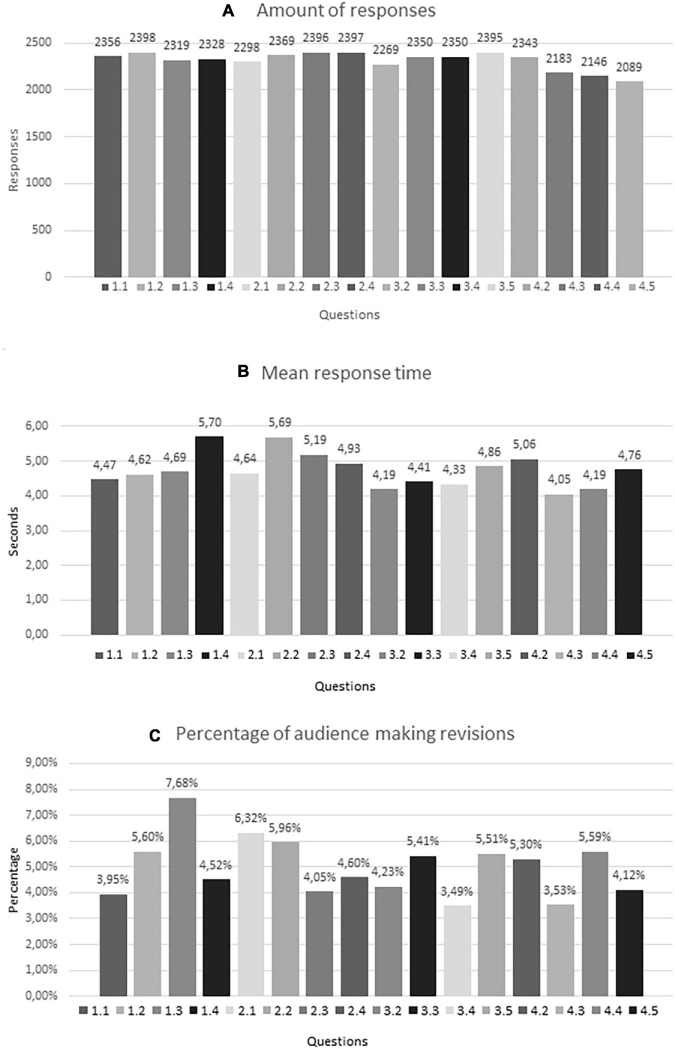
Interactive questionnaire (interactQ): The graph shows: **(A)** the combined amount of responses for each question, **(B)** the mean response time in seconds to answer the questions, where the *Y*-axis represents the response time in seconds and the *X*-axis represents the 16 questions, and **(C)** the percentage of audience members who made one or more revisions of their answers to the questions, where the *Y*-axis represents the percentage of the audience who made one or more revisions and the *X*-axis represents the 16 questions. For technical reasons we only differentiated between whether audience members made any number of revisions and if they did not. Two questions (3.1 and 4.1) were removed from the analysis as the majority of responses for these were lost due to technical issues.

Figure 4 shows the implicit measures of engagement from the interactQ with number of responses (4a), the response times (4b) and number of answer-revisions made (4c). The figure shows that out of the total audience sample (*n* = 2,604), there was an average of 2,312 answer-responses across all questions of the interactQ, with a slight drop in responses at the very end. The audience used an average of 4.73 s in mean response time with a maximum difference being on average 0.68 s. quicker (4.04 s) for question 1.4 and 0.97 s. slower (5.70 s) for question 4.3. In average 5.02% of the audience (116 people) revised their answers, with a maximum of 7.68% (178 people) for question 1.3 and a minimum of 3.53% (82 people) for question 4.3.

Table 2 presents the descriptive data given by the focus-group in the phenQI and the analytical categories. Here a selection of quotes illustrates the 29 tags used in the qualitative data analysis, 9 themes developed from these, as well as three overall categories (intervention, nuancing and reflective effect and attitude formation). The interviews show that participants experienced an awkward and biased first encounter with JN, which they describe as instinctive and relate to being prejudicial (quote 1A). They describe their initial attitudes as objectifying toward JN, as well as based on false beliefs about what it means to be physically disabled and how this affects JN, e.g., that he has a low IQ.

**TABLE 2:** Phenomenological qualitative interviews (phenQI)^a^.

Theme	Codes	Descriptions
**1. Category: Intervention**		
	Reaction	1A: “I don’t like to think of myself as prejudicial, but the play makes you recognize that JN is mentally, humoristic, and personally like everyone else, and that it is so unfair that you instinctively have awkward or reserved reactions when you meet him.”
	Meeting	1B: “You meet JN, you hear his story, and himself telling it. That makes a huge difference…It makes more of a difference than some campaign, where someone tells about how they are. It is not until you are confronted with it that it affects thoughts and understanding.”
Situated	Presence	1C: “Theater is in your face and you have to figure out your attitudes instantaneously. And you have to form the attitudes even if you haven’t thought about it before. The same would never happen with a newspaper or a video on facebook. In that case, you would not have seen it nor thought about it.”
	Focus	1D: “Theater is an intense way to concern oneself with a topic, whereas in articles you already have to agree with the premise and argument before you engage. It is rare that you tell yourself: now I will really try to put myself in the shoes of, and understand, that person.”
	Emotional	1E: (1) “It’s not intellectually and politically correct knowledge you get. You could call it experience or bodily knowledge” (2) “There are so many facts today, also with the debate of the post-factual society, which drowns the topic. But when it’s a human of flesh and blood and emotions, it is different.”
Bodily	Empathy	1F: (1) “It is something about feeling the challenges that they have, to get it under your own skin.” (2) “I don’t know how it feels. I have not experienced it and therefore I feel that I cannot put myself in the shoes of how it feels, but [in the theater] I tried to.”
	Action	1G: “By having one actively and physically do something, it forces you to form attitudes, rather than sitting still and thinking about the things being said.”
	Sharing	1H: “I think the theater gives you something exceptional, compared to other media. It is so present. It’s here and now. It is synchronal. We experience it together and we share it. It’s an intimate space you don’t get with a book, or a film, or a newspaper.”
Social	Group	1I: “I was outraged by the answers of the audience, or at least baffled. I simply couldn’t comprehend some of the answers. But I didn’t want to navigate along the majority. I want my own attitudes and opinions.”
	Atmosphere	1J: “The atmosphere was pretty intense, and you were forced to see and react to some things, which I normally don’t do.”
**2. Category: Nuancing and Reflective effect**		
	Understanding	2A. “To meet the person in this way and get a better understanding of his internal life, and how he experiences the world. That has definitely given me a better understanding.”
Knowledge	Depth	2B: “It’s the nuance and the depth of understanding it, that is crucial”
	Facts	2C. “I think the play gave me the knowledge to answer questions about disability based upon a large basis of factual insight.”
	Graduation	2D: “There are degrees to disability. It’s a spectrum and you can be different in many ways.”
Complexity	New Beliefs	2E: “People with disabilities are not unintelligent, which is new to me.”
	Neither/Nor	2F: “I want to be neither positively nor negatively discriminating”
	Perspectives	2G: “Seeing the others’ answers didn’t affect my personal answer, but it gave different feedback to them. You have one perspective and then you see others answer something very different. It helps to expand you mind, that not everyone thinks the same. Everyone has different attitudes.”
Openness	Inclusiveness	2H: “It is all about ignorance and I left the theater with greater understanding and scope than I went in with.”
	Personification	2I: “It is happening right in front of you, seeing him, and seeing that he is a real person. It becomes something that you can relate to.”
**3. Category: Attitude formation**		
	False beliefs	3A: (1) “I though spasticity was both physical and mental” (2) “As a society, we don’t know how to meet [people with disabilities] where they are, but we get stuck, and treat them as a thing or something that can’t think.”
Self-critique	Prejudice	3B: “I’m uncomfortable being around physically disabled [people] because I probably don’t understand them. I have a prejudice about it being both physical and mental issues. So, you have to get to know them.”
	Embarrassment	3C: (1) “I was actually embarrassed by myself” (2) “I was embarrassed about the way I have been thinking about people with disability.”
	Normality	3D: “You experience him as totally normal, as he is like everyone else. The things that he dreams about are things that we all dream about.”
Self-conception	Humanization	3E: “How can we judge him, just based on how we experience his physique? Because he’s also just a human being. We need to see the human first and then everything else.”
	Indentification	3F: “I heard the main question in the theater as: Do you ever feel alienated by others? And yes, I do, because I can identify with [feeling alienated].”
	Forming attitudes	3G: “I have never thought about these things before, so now I actually have reflected upon the things, which gives me a ballast to evaluate what I mean by disability.”
Self-correction	Categories	3H: “I start to think about and rethink what I have learned to categorize as disability, which is part of how I understand myself. This is the way that I categorize the world.”
	Self-evaluation	3I: (1) “There are things you realize…When you sit and think about it you can find out that you are still the person you are, or you can find out that you are actually more disgusted by disability than you will admit.” (2) “I made an evaluation of myself and relate to myself, and then I related that to the others.”

*^a^Table 2 shows a schematic account of the descriptions given by the participants in the focus-group that participated in the interviews. The table show different examples of descriptions that were the basis for an initial conceptualized coding of the descriptions. Each code are categories under a master heading relating to the intervention, the nuancing and reflective effect, and the attitude formation.*

However, they also show that the participants (re-)form attitudes toward JN during the intervention in an attentive and reflective way, increasing awareness of their own attitudes (quote 3G, 3H). Participants mention the performance as a source of new and factual knowledge about people with physical disability (i.e., out-group). It is a gateway into a deeper understanding of the person (in this case JN) behind the category “physical disability” and of the degree of complexity involved in belonging to this group (i.e., personalization). Noticeably, some interviewees particularly mention a difference in receiving this information and knowledge about physical disability within theater, as compared to other media (quote 1C).

Interviewees indicated that the social and immediate nature of the theater situation influenced the attitude formation through a sense of belonging here and now to a shared space (quote 1H). They further mention that being directly addressed and experiencing the explication (some particularly mention the interactQ) make them become aware of their own prejudice and beliefs about people with physical disability and the variety of attitudes it is possible to hold (quote 2G). Participants also describe that this self-reflectivity made them feel embarrassed and uncomfortable (quote 3C). Yet it also made them normalize and humanize the person with physical disability, to the point of personal self-identification (quote 3D). Participants described that such identification elicited a further self-reflection process – making them reflect on how they typically form attitudes, what kind of categorizations they typically use to form attitudes, and how they should evaluate themselves in such processes of attitude formations in the future (quote 3I).

## Discussion

In this section we discuss the results. The results indicate that we achieved the engagement aimed for in the experimental setup, and we note that there were significant differences between groups in the QQ. However, the difference is not unequally one of moving toward more positive attitudes. Further, there was no change in implicit attitude measure (IAT). We draw on the results from the phenQI to make sense of these findings, and suggest first that we obtained a nuancing effect, still countering stereotypical views on people with disability, and that this effect occurred primarily as an effect of second-order attitude formation (i.e., increased awareness on own formation of and reasons for forming attitudes). To capture these findings, we propose an Enact framework as an alternative to the more established contact theories. We end with some critical remarks on strengths and limitations of our methodological setup.

### Engagement and Attitude Change

Firstly, we wished to make sure that the performance succeeded in creating engagement – as defined by both experiential and normative aspects. The phenQI interview show that the encounter is characterized by the interviewees’ initial awkward, uncomfortable, and reserved, objectifying and prejudicial attitudes toward JN. These results replicate existing research on attitudes toward people with physical disability, as described above: the immediate feelings that people without disability experience are that of discomfort and fear, based on the objectification and perception of people with disability as incompetent. Such attitudes are associated with intergroup anxiety and known to decrease engagement.

However, the interviewees describe that as the performance progresses, they acquire a situated, intimate, and bodily understanding of JN, i.e., him as individual, rather than as an abstract disabled body or object; they commit to him, and he commits to them by being right there in flesh and blood, in their face, and telling his personal story. PhenQI thus affirms that the performance succeeded in giving the focus-group a situated, bodily and emotional understanding of living with a physical disability, as well as a normative commitment between audience and JN, despite initial awkwardness. In other words, members of the audience – sampled through the interviewees – experienced a second-person engagement with JN. Interviewees also indicate that the embodied and shared space of the theater is an important element of this engagement, supporting the claims of the second-person methodology made in the introduction.

Additionally, Figure 4 shows that in answering the interactQ, a high number of audience members were consistently active during the performance. With a few exceptions, and with a minor drop toward the very last part of the performance, their responses, their response time, and the amount of audience members who made answer revisions were consistent throughout the performance. This indicates that the audience’s commitment and active interaction with the questionnaire was intact during the performance, which are both signs of engagement and would not have been expected, if engagement had been absent.

Based on these results, we conclude that the performance kept the audience committed and engaged with JN in the intervention. In addition, the results of the phenQI indicate that the engagement has an effect on the audience’s attitude toward JN and physical disability. This is corroborated by the results of the QQ, which show a change of attitudes between pre-QQ (group 1) and post-QQ (group 2), when compared to the control group. This signals a reduction in prejudice regarding the specific aspects and situations of social distance and incompetence. Although this could be explained by selection bias, we see a significant tendency for improved attitudes between pre and post, indicating an effect of the intervention, nonetheless.

### Re-defining Prejudice Reduction: The Nuance and Self-Reflection Effect

To our surprise, the QQ results also show some effects of a decrease in positive attitudes, which could signal increase in prejudice, i.e., post-QQ participants are less positive than the pre-QQ participants – although not significantly. Initially we considered this a problematic outcome, since we expected that a reduction in prejudice toward people with physical disability would be a change from negative to more positive attitudes when it comes to competence and social distance. However, the reports of the phenQI indicate that through the engagement of the performance, the interviewees start to experience and understand the complexity and individuality of living with physical disability. Their attitudes and understanding of disability therefore become increasingly nuanced and less stereotypical.

Research shows that the prejudice that exists toward people with disability can be characterized as “paternalistic prejudice,” where both positive and negative stereotypes are mixed into the prejudice. This means that people without disability perceive people with disability as incompetent, they fear, objectify, and pity them, while at the same time showing compassion, sympathy, and even tenderness toward them (Fiske et al., 2002; Coleman et al., 2015). A nuanced experience and understanding of JN and people with physical disability, would therefore reduce prejudice by reducing negative attitudes of e.g., incompetence and social distancing, while at the same time reducing positive, stereotypical attitudes that leads to pitiful compassion and sympathy toward people with disability.

Our proposal is thus to see prejudice reduction as a nuancing of attitudes rather than mere change in positivity, or in other cases, mere negativity. This conception of prejudice reduction is especially relevant in the case of physical disability and other groups where paternalistic prejudice is dominant. We thus propose that when reporting prejudice reduction, one might aim at measuring a nuancing effect, rather than a positive/negative improvement. However, this proposal and the connection between prejudice, nuanced attitudes, and measures should be investigated further.

In the phenQI the participants also emphasize their relation to the rest of the audience (in-group relation). Answering the interactQ, they are forced to form their attitudes about physical disability and JN, and their evaluations are shown in real-time on stage, making it visible to JN and contextualized in relation to the replies of others. This means that they see how their individual evaluations correspond to the majority or minority of the audience group. Such comparison gives the participants additional reflections and perspectives on their own attitudes in a social setting. This is for instance, seen in reports of feeling embarrassed about their reply, which most prominently happened when interviewees realize that their answers were stereotypical and not supported factually or were part of the minority of answers from the audience.

The interactQ which was developed to implicitly measure indicators of engagement thus turned out to be a key feature mentioned in the self-reported prejudice reduction, for the attitude explication and social contextualization that it provided. The focus-group interviewees report that the nuancing effect of engagement with the out-group member, and the social effect of the real-time interactQ, make them self-reflectively aware of their own prejudice, and stereotypical beliefs about people with physical disability. They become self-reflective about the act of forming their attitude, which means reflecting on why a possible dissonance (e.g., embarrassment) occurs. This self-reflection enables them to answer the questions of why they hold the attitudes they do, rather than merely causing them to correct their attitudes.

The prejudice reduction we see can therefore be explained as reduction on an explicit and conscious level, rather than implicit level. It concerns increased engagement with the attitude object (i.e., people with physical disability), which creates a nuancing and self-reflective effect. This may explain the lack of significance in the IAT test results. The effect of the intervention would not show up on a binary (good-bad) IAT test, since such tests target “automatic” and “implicit” associations that operate at a lower (un)conscious level of attitudes. Whether the engagement-based strategy for prejudice reduction can have effects on the implicit level would require further research.

Given this discussion we expand our initial proposal of reducing prejudice through an engagement-strategy with two further points: nuance and attitude formation. We formulate this in the *Enact* (Engagement, Nuancing, and Attitude formation) hypothesis, which posits that interventions working with an engagement condition can reduce prejudice through a nuancing effect and by working with a second condition focusing on the formation of attitudes. This also means that we redefine the aim of prejudice reduction, which should not be thought of as changing either positive or negative attitudes and stereotypes, but as a nuancing of attitudes and stereotypes.

### Enact vs. Contact

The risk of backfire effects (e.g., intergroup anxiety, disengagement, and reinforced prejudice) is particularly known in attempts of attitude correction (Campo and Cameron, 2006; Nyhan and Reifler, 2010). In the Enact hypothesis, we aim to avoid the risk of backfire effects, not by adding conditions to the list of direct contact or by removing the direct embodied contact, described in the introduction as strategies used by current versions of contact theory, but by distinguishing *attitude correction* from *prejudice reduction*. As we saw with the results, attitudes may go both ways at once, so to speak. Reducing prejudice or stereotypes, as we see it, is therefore not a question of improving categories, but of de-reification (Berger and Pullberg, 1965). That is, de-reifying orthodox frameworks for knowing and relating, or as formulated by Allport “a differentiated category is the opposite of a stereotype” (Allport, 1954).

Even without the attempt to correct the audiences’ attitudes, we saw in the results from the phenQI that the audiences experienced embarrassment in their engagement with JN. As described above, these negative emotional experiences of discomfort are usually to be avoided in direct and indirect contact approaches, as they are related with intergroup anxiety and pose a risk of disengagement and stereotype strengthening. In our intervention, however, the interviewees do not focus on ways of realigning cognitive content and escape embarrassment, but rather on how they reflected on the embarrassment. As shown in Figure 4, only few participants (in average 5.02%) revised their answer during the performance. We take this as an indication that the audience took their time to consider their answer and stuck to it, even if they would be embarrassed by it. Dealing with intergroup anxiety through self-reflection is a possible explanation for why the reports of initial embarrassment in the encounter did not lead to disengagement, as could have been expected.

To emphasize this effect of self-reflection, we draw attention to the second-order level of attitudes, namely the act of attitude formation, rather than the first-order level of attitudes, i.e., the content of the specific attitudes. In contrast to the direct and indirect contact approaches, results indicate that the intervention did not primarily correct or change attitudes at the first-order level. Instead, the participants became aware of the second-order act, i.e., aware of how and why they (re)form their attitudes when they engage with the attitude object (e.g., JN and physical disability).

This means that in the Enact hypothesis the aim is for people to self-validate their own attitude formation, rather than to increase informational content about the attitude object. Recent persuasion research has defined self-validation as a parameter associated with high credibility to the attitude source, and as a parameter which generates both personal relevance and self-confidence in the attitude formation (Briñol et al., 2009). With an attitude formation based on a nuancing process, the changes will originate from the participants themselves – not from external factors – meaning that they will entail self-validation, which provides a certain strength to the attitude (re)formation.

This may also address the issue of long-term exposure as seen in some contact interventions, i.e., the assertion by Pettigrew (1998) that content-related attitude change often demands repeated and frequent direct or indirect contact. We suggest that this may not be the case in second-order attitude change. Since the engagement strategy effects changes on the structural level of forming attitudes, there might not be a necessity for the same degree of repeated direct or indirect contact. Interviewees did mention that they returned to the experience in the theatre in the days after the performance when they encountered other people with physical disability at workplaces or in public spaces. However, we did not measure for such long-term changes (see strengths and limitations section).

How does the Enact hypothesis relate to the direct and indirect contact hypothesis and does it solve the application challenge? To deal with the application challenge the Enact hypothesis operates in a hybrid way that includes and combines aspects of both direct and indirect contact strategies. The Enact hypothesis is thus a version of contact theory, working with contact in terms of engagement^7^.

To deal with the application challenge, the Enact hypothesis posits two necessary conditions for prejudice reduction: (1) engagement, and (2) explicit reflection of the attitude (re)formations. However, drawing on theater as the intervention for contact implicitly controls further factors of the contact. In the use of theater, the settings, customs, and norms of the theater regulate the contact, as they structure the engagement between the in- and out-groups, e.g., the audience voluntarily agrees to see this performance, and it is legitimated by an institution. Theater was chosen because it operates with shared, embodied participation between in- and out-group, but placing JN on stage further effects this interaction to the end that the out-group is granted higher status (audience is listening to JN’s experience). This means that depending on which intervention one uses additional factors and structures may help in structuring the contact^8^. It nevertheless shows that using theater as an intervention is one way to create engagement, nuance and reflection, and thereby to reduce prejudice in a real-life setting.

Finally, the phenQI results highlight that the current social media, information, and technological landscapes are determined by potential disengagement. Thus, the use of those strategies in solving the applicability challenge (particularly for their benefit of vast scalability) comes with a risk of disengagement, and from that with a risk of reinforcement of stereotypes, as outlined in the introduction. Since engagement is a necessary condition for reduction of prejudice in the Enact hypothesis, one must be aware when choosing and designing the intervention medium that engagement is possible and ensured, and preferably monitored by indirect and direct measures.

### Strengths and Limitations

We conceive of this study as an initial exploratory phase of developing a hybrid strategy for prejudice reduction. We therefore maintain that there are good indicators that the Enact hypothesis has something to offer, and that further investigation and development is warranted. Here, we explicate a few of the method-implications for the strength of the results.

In terms of evaluating selection bias, we draw on statistical material from general population as well as theater audiences. Of the seven groups involved in the study (see Figure 1) we have biographical information on the four pre- and post-groups of the audience (group 1 + 2 + A + B), as well as on the focus-group. Those can be compared to data about the general population, as well as data on the average theater audience in Denmark (see Table 1). We do not have data on the general audience group (included in the interactQI), and only limited data on the control-group, showing a slight variation in gender and age to the general population (see Table 1). It should be noted that the test-selected groups (group 1 + 2 + A + B) and main part of the general audience group were self-selected by buying tickets for the performance, i.e., knowing the title and general topic of the performance. The focus-group was on the other hand randomly recruited and invited to the performance. Although they had to agree to go to the theater they were not informed of the title nor of the topic of the performance.

In the focus-group we see a predominance of women compared to men, mirroring the distribution for the average theater audience^9^. However, in the test-selected groups, the ratio is reversed with a dominance of male participants. Both groups, focus- and test-selected, show a younger average age than the median age for average theater audience and they also show higher levels of education than both the average Danish population and average theater audience. The latter might be explained by the lower average age, as the level of education is higher among younger generations (Nørtoft, 2014). Further, the performance took place in traditional theatrical institutions tied to higher social class. The participants might therefore had been different had we used community theater settings or street performance. The age difference may indicate that our intervention performance has attracted a slightly different crowd than usual theater that attracts on average an older population.

There are two things to note from this. First, there is some selection bias (whether self-selection or recruitment bias). We have tried to balance this by also drawing on a control-group, and by recruiting groups from two different settings: the audience and a randomly recruited focus-group in the city center. Some selection bias is likely to be shared by these groups, e.g., an interest in theater^10^, but other elements will vary. Second, levels of education might be a particularly important factor in the outcomes of our study, and further studies should be carried out to test the Enact-interventions in more diverse, cultural settings.

On average, 10–15% of the Danish population has a disability. The focus-group participants have a lower percentage, whereas the test-selected groups place themselves in the high end of the spectrum (14.7%). There is no data available on disabilities in theater audiences. Approximately half of both groups know someone with a physical disability. We do not have a specification of the closeness of the relations, and there is no data available for this number in the general population, nor in general theater audiences. However, assuming that these numbers are indicative of the general audience, this means that even if there was self-selection of audience members – risked by the general audience knowing the topic in advance – approximately half of the audience did not have prior acquaintance with someone with physical disability, and thus, at least self-selection of in-group or in-group familiars did not occupy the entire test group.

As for possible transfer between different target out-groups (i.e., race, gender, sexuality or other groups subject to prejudice) we see no theoretical limitations for the transferability of the Enact hypothesis. However, Paluck et al. (2018) finds evidence that contact seems to be particularly effective for prejudice reduction in the case of physical disability. This may also apply to our hypothesis, and the tendencies we have shown may not be as present in other target groups.

An unanswered question in relation to our results is whether it generates long-lasting effects of prejudice reduction. It would be interesting for future experiments using the Enact hypothesis to do cross-sectional and longitudinal follow-up studies of individual participants, to see if immediate attitude change could become long-term and potentially change the behavior toward people with physical disability or other out-groups. In this experiment we did not conduct such studies, but we conducted interviews 4 months after the pilot-experiment, which indicated behavioral changes in relation to the participant’s openness when meeting people with physical differences in their daily life.

One thing to consider in new Enact-interventions is an attentiveness to unexpected ethical challenges due to the effect and manipulative skill of the theater performance, namely the transformative effect that lies in engagement. In one case, a focus-group participant was clearly upset after the performance and told a member of the research group that they found the intervention very uncomfortable and did not feel they had properly agreed to having their attitudes and beliefs challenged. However, as it might affect results in multiple ways – e.g., creating selection bias and priming test participants – to fully inform participants beforehand, an ethical dilemma arises. Although such strong discomfort should not be inflicted on test participants against their will, we suggest that the best responsible handling of this is through a proper off-boarding process. In our case, we made sure that research and theater personnel were present and clearly visible when the audience exited the performance. If any audience member wished or felt a need to discuss the performance, they were able to make contact and research or theater members would listen, answer questions, and make sure that no one left the location feeling distressed.

## Conclusion

In this paper, it has been our aim to deal with the application challenge of the contact hypothesis, by using a hybrid strategy of both direct and indirect contact. Based on second-person methodology, we developed an engagement strategy for prejudice reduction. We implemented the strategy in a theater performance and investigated the effects on prejudicial attitudes toward people with physical disabilities. From the case study results we reformulated our initial engagement strategy to an Enact (Engagement, Nuancing, and Attitude formation) hypothesis. This hypothesis deals with the application challenge by positing two necessary conditions for prejudice reduction. Interventions should: (1) work with engagement to reduce prejudice, and (2) work on the second-order level of forming attitudes. However, working with such a strategy, means that the goal of the prejudice reduction should not be thought of as attitude correction. Instead, the aim for reducing prejudice should be to nuance attitudes. We see good indications for further development and investigation of the Enact hypothesis.

## Data Availability Statement

The original contributions presented in the study are included in the article. Additional data can be found at the Open Science Framework webpage: https://osf.io/n32zu/.

## Ethics Statement

Ethical review and approval was not required for the study on human participants in accordance with the local legislation and institutional requirements. The patients/participants provided their written informed consent to participate in this study. Written informed consent was obtained from the individual(s) for the publication of any potentially identifiable images or data included in this article.

## Author Contributions

KM: conceptualization, methodology, investigation, supervision, project administration, and writing – original draft. HS-F: conceptualization, methodology, and writing – original draft. AJe: software, formal analysis, data curation, and visualization. AJu: methodology and formal analysis. DN: investigation and formal analysis. TC: resources, project administration, and funding. All authors contributed to the article and approved the submitted version.

## Conflict of Interest

The authors declare that the research was conducted in the absence of any commercial or financial relationships that could be construed as a potential conflict of interest.

## Publisher’s Note

All claims expressed in this article are solely those of the authors and do not necessarily represent those of their affiliated organizations, or those of the publisher, the editors and the reviewers. Any product that may be evaluated in this article, or claim that may be made by its manufacturer, is not guaranteed or endorsed by the publisher.

## Footnotes

1 The pilot-experiment and its results are discussed in Martiny (2015).

2 The authors assert that all measurement procedures contributing to this work comply with the ethical standards of the relevant Danish committees on human experimentation and with the Helsinki Declaration of 1975, as revised in 2008. Ethics approval is not obtained for this experiment because, according to the Danish ethics committee law §2, no. 1, only health scientific research that includes biological material and clinical trials needs approval, whereas questionnaires, qualitative interviews and non-health scientific intervention studies, do not need approval.

3 See Martiny et al. (2021) for at further description and discussion of the phenomenologically based mixed-method design.

4 For further description of how to conduct mixed-method triangulation with a phenomenological frame see Martiny et al. (2021).

5 The data and results that were not included in Figure 2, along with additional material (e.g. the questionnaires used), can be found at the Open Science Framework webpage: https://osf.io/n32zu/.

6 The scale also includes slight, moderate, and strong preference *for disabled*, but as no participant achieved this result, these preferences are not included in the ranked data.

7 Thanks to reviewer 1 for clarifying this relation between the Enact and Contact hypothesis.

8 Thanks to reviewer 1 for clarifying that the Enact hypothesis does not only work with two conditions, but depending on the intervention it works with, additional factors and structures can be added.

9 It should be noted that the focus-group consists of a relatively small number of people, skewing the biographical statistics by 3.33% for each participant.

10 It could be argued that such a self-selected group may be more easily engaged than an average population. We do not deny this. Our intervention test focused on whether there would be an effect, given engagement was present. Underlying reasons for the engagement are thus interesting, but not directly relevant.
